# A preparedness model for the provision of oral health care during unfolding threats: the case of the covid-19 pandemic

**DOI:** 10.1186/s12903-021-01627-8

**Published:** 2021-05-12

**Authors:** Mario Brondani, Leeann Donnelly

**Affiliations:** 1 Director - Diversity, Equity and Inclusion; Chair – Dental Public Health, Department of Oral Health Sciences, 116/2199 Wesbrook Mall, Faculty of Dentistry, University of British Columbia, Vancouver, V6T 1Z3 Canada; 2 Director - Community Engagement; Department of Oral Health and Biomedical Sciences, Faculty of Dentistry, University of British Columbia, Vancouver, Canada

**Keywords:** COVID-19, Qualitative research, Oral health, Canada, Dental model, Preparation

## Abstract

**Background:**

The aim of this study was to appraise a recently developed preparedness model for the provision of oral health care during a threat such as the COVID-19 pandemic from the perspectives of oral health care providers, administrators/staff, and patients.

**Methods:**

An exploratory qualitative inquiry via at-a-distance semi-structured interviews and group discussions engaged a purposefully selected sample of oral health care workers and patients in British Columbia (BC), Canada. Participants were asked to appraise a preparedness model by considering *how to prepare for oral care during a pandemic*, while answering open-ended questions about the model content and visual presentation. Interviews and group discussions occurred between April 2020 and January 2021, were audio recorded, and transcribed verbatim. An inductive coding process was used to identify themes, subthemes, and categories of information until saturation was achieved.

**Results:**

Seventy-four participants, including 19 dentists, 15 dental hygienists, 10 certified dental assistants, 9 administrators, and 21 patients, suggested modifications to the recently developed preparedness model. Individual interviews (41 participants) and group discussions (33 participants in groups ranging from 2 to 9 attendees each) lasted for an average of 53 min. Eighty-four hours of audio recordings led to more than 1110 single-spaced pages of transcripts. The thematic analysis identified 82 codes, 12 categories, and four main themes: life-long learning, critical thinking, personal and professional risk, and patient-centred care. These themes were understood within provider characteristics and social and environmental contexts. Participants highlighted the need for the model to focus on information and communication, developing awareness and understanding, inferring risks, and performing oral health care during a threat such as a pandemic or disease outbreak. A modified portrayal of the model was suggested to better represent participants’ perspectives.

**Conclusion:**

A recently developed preparedness model for the provision of dental care during an unfolding threat like the COVID-19 pandemic was appraised and modified by oral health care workers. Future studies are warranted to evaluate the modified model for use in the event of another unfolding threat collaboratively with providers, patients and stakeholders.

## Introduction

As of May 1 2021, the COVID-19 pandemic, caused by severe acute respiratory syndrome coronavirus 2 (SARS-CoV-2), has spread to more than 200 countries and territories, caused over 3 million deaths, and infected more than 150 million people worldwide [1, 2]. Also by May 1 2021, a third wave of infections was experienced by a number countries, some caused by more infectious genetic SARS-CoV-2 variants [3], even in the wake of mass inoculation efforts made possible by the fastest development of a vaccine ever seen in modern history [4]. Nonetheless, the pandemic continues to lead to social unrest [5] and economic and educational pitfalls [6]. The pandemic has also negatively impacted the provision of health care, and in particular oral health care, due to the close face-to-face proximity of professionals to patients’ face [7]. As the virus that causes COVID-19 can be found in saliva droplets and aerosols, the practice of oral health care is said to be at the highest risk for transmission of the virus [8, 9] even more so in light of a strong evidence for airborne spread as discussed by Greenhalgh and colleagues [10]. The current infection control strategies, introduced during the HIV/AIDS era [5], have been enhanced, albeit inconsistently, in many oral health care protocols during the pandemic [11, 12].

Despite oral health care providers being prepared to mitigate the daily risks of known pathogen transmission in their practices, the emergence of SARS-CoV-2 initially halted the provision of care [13, 14]. The subsequent pandemic reopening plans varied across different countries, and even within the same country [11], and were likely a reactive response lacking a more agreed upon approach [15]. Such variation might have been caused by the absence of an available preparedness strategy or model applicable to oral health care to proactively deal with SARS-CoV-2 infections [16]. Previously, preparedness models have been suggested for use during threats of infectious disease outbreaks such as the Ebola pandemic [17] In general, preparedness encompasses the planning and responses to terrorists attacks and environmental (e.g., earthquake, cyclones, tsunamis) or health disasters (e.g., infections such as Ebola, SARS, and the Middle East Respiratory Syndrome) [18]. In particular, preparedness can be understood as *“*the knowledge and capacities … to effectively anticipate, respond to, and recover from, the impacts of likely, imminent or current hazard events or conditions” (p. 21) [19]. Such preparedness requires the development of communication plans and collaboration among different institutions, organisations, and levels of government, while remaining flexible and adaptive to changes, particularly in the event of an infectious outbreak. Without a preparedness model to aid in understanding the threat posed by the COVID-19 pandemic, uncertainties were experienced despite information being available from studies on readiness to deal with medical emergencies [20, 21] and requirements for special needs patients in dental offices [22]. As such, this study aimed to appraise a recently developed preparedness model for the provision of oral health care during an unfolding threat such as the COVID-19 pandemic from the perspectives of oral health care providers, administrators/staff, and patients. In order to achieve this objective, an exploratory qualitative study was suggested to better facilitate the model’s appraisal shaped by open-ended questions. Qualitative studies are employed to gain a deeper yet subjective understanding of underlying reasons, opinions, and expectations about a given topic, and to tease out similarities and differences in participants’ values and beliefs [23]. Qualitative research employs a variety of inductive methods, from field observations to direct interactions with individuals or groups of participants. In dental research, qualitative inquiries have been used to explore how the COVID-19 pandemic has lead to uncertainties on providers [5] and students [6] how stigma has been experienced by those living with HIV/AIDS [24, 25], how certain populations access oral health care when encountering adversities [26, 27], just to name a few. This study is the outcome of a larger investigation, with the overall goal to develop and assess a preparedness model that can aid in guiding individuals to make sense of the situation at hand by looking at the available information [5, 6, 12].

## Methods

An exploratory qualitative inquiry employing semi-structured at-a-distance interviews and group discussions engaged a purposefully [28] recruited sample of oral health care workers (e.g., dentists, dental hygienists, certified dental assistants, front desk staff, and administrators) and patients from across British Columbia, Canada. Approval for this study was obtained from The University of British Columbia’s Behavioural Research Ethics Board (# H20-01147). A qualitative inquiry took place between April 2020 and January 2021 to gather information on the curtailment of oral health care services in light of the COVID-19 pandemic; this timeframe covered the provision of emergency care (first wave of infections; March–July 2020; 45 interviews) and the resumption of regular care (second wave of infections; November 2020–January 2021; 29 interviews). As in our previous studies [5, 29, 30], participants were informed about the research via an email distributed to a province-wide professional list, and through snowball sampling via word-of-mouth. Inclusion criteria covered any potential participants who were unemployed (e.g., offices or practices were closed—oral health care workers, patients who had been laid-off, etc.) or continued to work on a full- or part-time basis, those who were of any gender, and those older than 19 years of age. For the oral health care providers, we attempted to establish a somewhat even representation of professional roles (e.g., dentists, dental hygienists, certified dental assistants, and administrative/front-desk staff), years of experience in that role (e.g., less than 10 years and more than 10 years), and timing of the interview (during the first or second wave of infections) as previously suggested [5]. For the patients, we attempted to engage with those who were currently receiving oral health care and those who had not received care since the start of the pandemic.

Participants contacted the first author via email, who then emailed them back with information about the study, the interview and group discussion process, and a copy of the informed consent form to be signed and returned. The informed consent also explained that the information gathered would be de-identified to maintain confidentiality and anonymity. The interviews and group discussions were conducted at-a-distance via phone or Zoom© video conferencing at a date and time convenient for the participants. Interviews and group discussions were conducted either by one of the authors who have extensive experience in qualitative studies [5, 24, 25, 33, 34] or by a hired research assistant who was trained at length by the authors; the interviewers were calibrated by interviewing the first two participants using a group format to refine the interview guide and set the pace of the interactions.

**Fig. 1:**
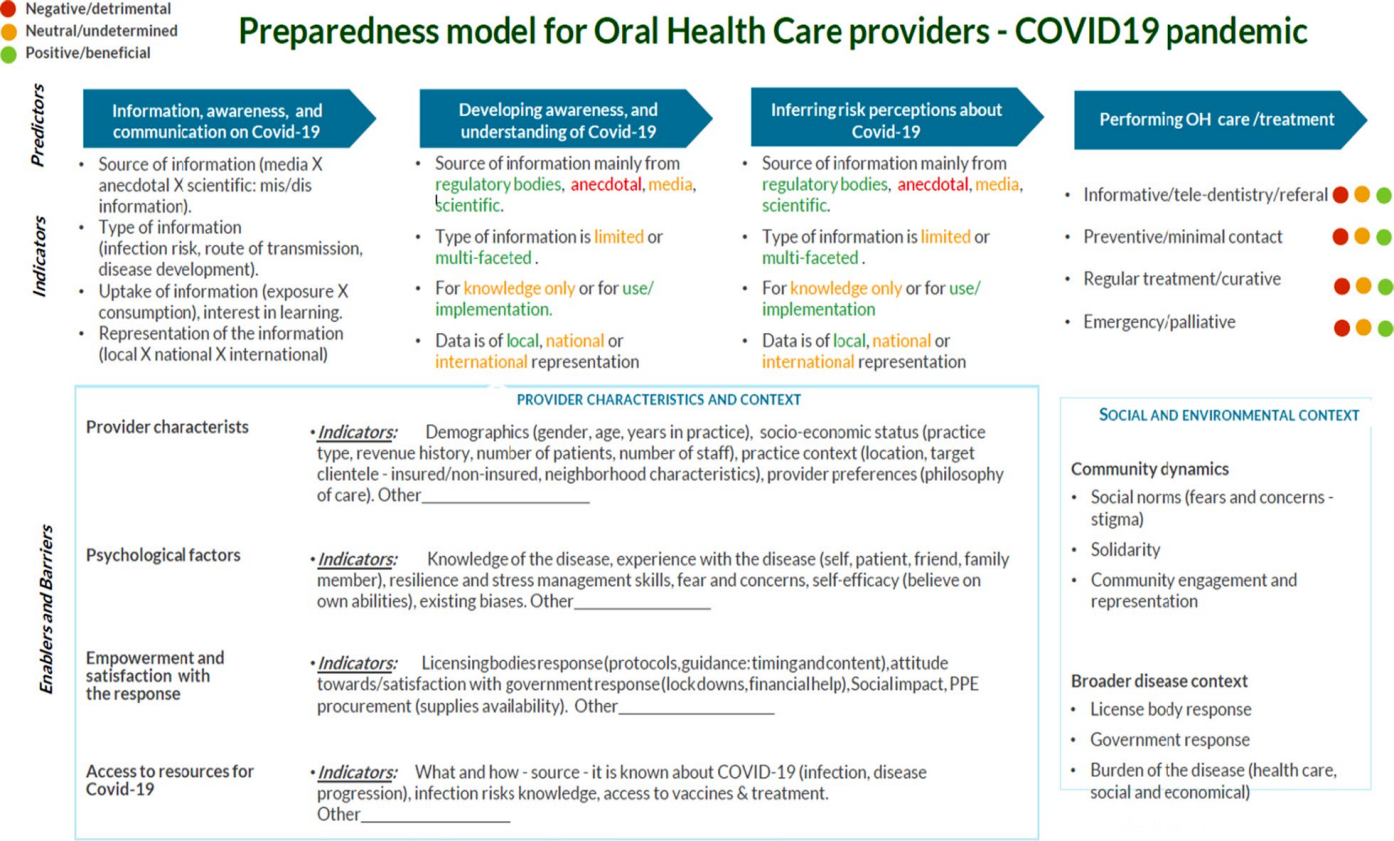
A preparedness model for the provision of dental care during the COVID-19 pandemic adapted from WHO’s Information Network for Epidemics

Participants were asked to appraise a preparedness model for oral health care adapted from the World Health Organization’s Information Network for Epidemics [31] (Fig. 1), while they considered *how to be prepared during a pandemic.* The model was focused on the type of information available, developing awareness about the disease, inferring risks of infection, and the decision to perform various levels of oral health care. It also accounted for provider characteristics, including enablers and barriers, and the social and environmental contexts, including community dynamics, that likely influence preparedness. The model also attempted to qualify the information about COVID-19 with a colour-coded scheme indicating positive/beneficial (green), negative/detrimental (yellow), or neutral/undetermined (red) information. Participants were given this explanation when shown Fig. 1 to minimise unintentional biases regarding its use, value, and applicability, and were then asked:

- What preparations during an outbreak are necessary to provide oral health care?
- How do you see this model being implemented and why?
- How can this model enable you to appraise the information about the pandemic?
- Given the different components in the model, including predictors, indicators, enablers, and barriers, how do you understand these components informing one another? Why?
- What do you understand by provider characteristics and context in the model? Is that important? Why or why not?
- What do you understand by social and environment contexts in the model? Is that important? Why or why not?
- What is missing from the model? Why?
- What should be removed from the model? Why?
- Is the visual representation clear? Why or why not?
- How would you rearrange its components? Why?

As in any qualitative inquiry, questions were posed loosely in the order above, and some might not have been explicitly asked if they were answered earlier on in the interview. Probing questions were added for clarification throughout all the interviews.

Interviews and group discussions were audio recorded, transcribed verbatim, and de-identified for interactive thematic analysis using NVivo® 12 software. An inductive coding process was used to identify the main themes of information from the transcripts related to the preparedness model presented, and to suggest modifications if a new portrayal was deemed necessary by the participants.

Coding refers to identifying specific ideas or labels (in the form of a word or words) within sentences or excerpts from the transcripts. The same sentence or excerpt can have more than one code. Related and similar codes are grouped under a specific category that encompasses all the codes deemed alike; analogous and linked categories are clustered together, representing a main theme. A qualitative study can generate a very large number of codes, a dozen categories and a handful of themes. This study opted for an inductive coding process grounded in the qualitative data itself—rather than a deductive process using a predefined set of codes—so that important ideas were not overlooked, as we have used extensively in other studies [32–34] Each participant received a $150 honorarium to acknowledge their contribution to the study. Rigour of the study was achieved by employing reflexivity [35] during data collection and analysis, reaching data saturation when no new information emerged and the data got repetitive [36], and conducting member checking back with the participants [37] in order to reduce subjectivity and attain the required standards for ethics and quality [38, 39].

## Results

### Participants

Seventy-four participants, including 19 dentists, 15 dental hygienists, 10 certified dental assistants, 9 administrators/staff, and 21 patients, were interviewed; 49 were female. As the participants reached out to the research team once they met the inclusion criteria, no participant declined to be part of the study. In fact, the number of participants who contacted the researchers was larger than the number who was actually invited to be interviewed given the principle of data saturation. The 53 oral health care workers varied in years of experience (from 19 months to more than 35 years) with 8 not having returned to work since the beginning of the pandemic (5 certified dental assistants, 1 dental hygienist, and 2 front desk personnel). All 21 patients were older than 19 years of age (mean age 38 ± 5.6 years); of these patients, 9 were men and 11 had not returned to see their oral health care provider since the beginning of the pandemic (data not shown). We recognised repetition of the information after 69 individuals participated; moreover, at least one additional interview with a representative from each participating group (e.g., dentist, dental hygienist, certified dental assistant, administrators/staff, patient) took place to ensure that saturation had been fully achieved. Individual interviews (41 participants) and group discussions (33 participants in five groups ranging from 2 to 9 attendees each) lasted for an average of 52 min. Eighty-four hours of audio recordings led to more than 1110 single-spaced pages of transcripts, which were then analysed thematically.

## Thematic analysis

**Fig. 2:**
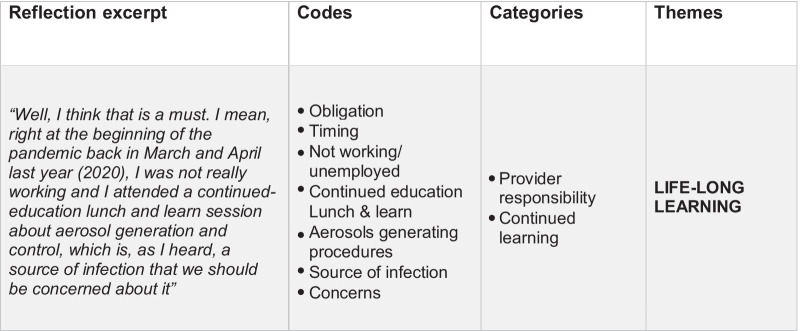
Coding scheme using an excerpt from an interview transcript in this study

The authors and hired research assistant calibrated their coding schemes using the first five transcripts. The authors separately coded a different set of 11 transcripts each, with the hired research assistant coding 35 transcripts (from five focus groups and 41 individual interviews). Although the coding was conducted independently, the three researchers met via conference calls to discuss the identified 82 codes, 12 categories, and main themes, and to reach consensus on the thematic analysis process. After coding the transcripts four themes emerged: life-long learning, critical thinking, personal and professional risk, and patient-centred care. As participants assessed the original model, they agreed with the language used in that portrayal (e.g., information, awareness, risks, etc.). Participants also believed that social and environmental contexts, along with provider characteristics should remain in the model, modified or not. Figure 2 shows an example of coding an excerpt from a transcript.

### Life-long learning

Participants were adamant in emphasising that the onus to learn and keep informed lays on the provider. The *“need to keep learning”* and *“continued-education”* were discussed by the providers themselves:

“This current pandemic is a classic example of the need to keep learning, reading about it, so we can get a sense of what is happening locally, nationally, and internationally” (35-year-old male dentist who returned to work after the pandemic).

“Well, I think that is a must. I mean, right at the beginning of the pandemic back in March and April last year (2020), I was not really working and I attended a continued-education lunch and learn session about aerosol generation and control, which is, as I heard, a source of infection that we should be concerned about it” (33-year-old female dental hygienist who was not working during the first wave of the pandemic, but still attended this lunch and learn session).

While the fact that providers should “*be on top of things*” was a “*no-brainer*” from the patients’ point of view:

“I would only expect that my dentist should be on top of things and get the latest and most accurate information about what is happening, and from good sources of information…that is their duty to us” (32-year-old male patient who had not seen an oral health care provider since the beginning of the pandemic).

“As somebody taking care of my health, and my teeth, it is a no-brainer that they [oral health care providers] have to know what is the best thing to do, and deliver the proper care to me … it is a constant learning process for them I think” (41-year-old female patient currently seeing an oral health care provider).

### Critical thinking

This theme emerged as some participants reflected on the development of awareness about COVID-19, particularly when asked how the model could help them navigate the pandemic. A currently working 41-year-old female front desk staff member commented, *“in order to make sense of the information that is out there, you have to be critical and ask yourself ‘is this possible? is this information correct?’, so you can evaluate what you are reading or listening to”.* In the words of a 39-year-old male dentist with over 12 years of experience, *“you develop awareness by understanding what is being said and by whom, so you can better appraise the whole situation … you think critically about it”.*

Various participants mentioned words like “critical”, “knowledgeable”, “judicial”, “rational”, and “unbiased” when referring to how one would develop awareness, and when considering the qualities of a good oral health care provider. In particular, a 37-year-old female patient who would be attending a dental appointment within 10 days of the interview pondered social media, TV programs, and awareness: *“I was flipping channels the other day and one of those programs pop up, that have a celebrity host …this time discussing the negative impact of lockdowns without referring to any source of information. It is scary to think that programs like this, much like the news from social media, are influencing peoples’ thinking”.*

### Personal and professional risk

Although participants did not question the implication of saliva droplets and aerosols on the transmission of SARS-CoV-2, the risk of infection was front and centre for virtually all those we interviewed. Patients, more than any other group, felt anxious about resuming their dental treatment, and many said they would wait to get vaccinated as they *“did not want to take any chances”*. Others, including a 33-year-old male patient who saw a dental hygienist 6 months after the COVID-19 pandemic was declared, felt it was a matter of common sense and hinted at the expectation that providers be on top of things, as mentioned earlier:

“Look, as a health professional I would only expect my hygienist to be protected and to protect me during the appointment…I had no problem in answering questions about COVID, in having my temperature checked, in wearing a mask until I sat on the chair ... and seeing her [dental hygienist] with that yellow suit-like covering, and a visor ... so we can all be vigilant against this disease and not make assumptions.”

Many of the oral health care workers were perhaps less concerned about the risk of SARS-CoV-2 transmission in a dental setting, given the usual universal precautions and the now heightened public health measures and use of personal protective equipment (PPE). In particular, a 39-year-old male dentist working in two offices for the past 13 years commented that *“we hear that we are at a higher risk of infection than others, but in reality the actual transmission is lower in a dental appointment, as I read”.*

According to a 43-year-old female certified dental assistant (not working at the time), the idea of risk and disease development surfaced while reflecting on the situation in nursing homes: *“I guess we all can get this disease, we all can be at risk, but look at the situation in many nursing homes … they got the disease and many died, their bodies did not fight back…it was sad”.*

### Patient-centred care

The idea of centering care around the patient’s wishes and preferences was highlighted by numerous participants when considering the nature of the treatment. We were told by a 32-year-old male patient, when looking at the modalities of treatment in Fig. 1, that *“now, more than ever, I want my dentist working with me and booking me in only if there is a need…like, if I’m in pain or have an infected tooth. If it is just to check things out or get a prescription, that would be totally fine over the phone”.*

The idea of tele-dentistry was received with mixed feelings. The exchange of ideas, presented below, took place between two patients being interviewed in a group. These two patients also discussed the issue of billing for an over-the-phone consult versus in person while considering the provider’s time needed in either situation.

P1 *“You are not going to the office in person, how is that the same as an appointment if they cannot see what is inside your mouth?”*

P2 *“Yeah, but I don’t think it would be for every case, I mean, sometimes you must go in person”*

P1 *“Exactly”*

P2 *“But what if we can show it with our cameras, from our phones…everybody has one. Wouldn’t that count as a quick check?”*

For a 35-year-old female dental hygienist with more than 10 years of experience and currently working, at-home prevention was highlighted: *“I may be seeing fewer patients now, but I get a sense that they are much more into their daily oral care at home, they are investing in that—last week I was booking a patient for a recall, and he declined while asking me which electric tooth brush to buy, and if a water flosser would help.”*

## A refined preparedness model

Very few participants agreed with original portrayal in Fig. 1 as initially presented. For those few participants who did, the model was *“extensive and with a lot of information … holistic but perhaps too much to digest and use*” (35-year-old female patient with an appointment booked to see an oral health care provider for the first time after the pandemic had been declared). Modifications were suggested from the other participants. One of the main points brought up was the fact that Fig. 1 did not account for dynamism when it comes to information development influencing previous steps, and the need to re-access a current or past situation:

*“*I see the predictors as good concepts, but how can it show the evolution or change of the information influencing them? I mean, if you infer the risk about an infection, you base [it] on the knowledge you have at the moment. But that knowledge may change as we know more. And that would impact the way we infer the risks. So, it has to allow us to go back and re-assess ” (38-year-old male dentist with 12 years of experience).

In turn, various participants suggested double-sided arrows or retroactive pathways to convey the idea of re-assessing a previous step based on new or modified information. For a 46-year-old female dental hygienist with 22 years of experience, *“I would draw arrows that allow you to go back a step or two and not only a one-way flow, as things change and what was true one day, might not be on another”.* A clear example of this change in information and knowledge was the use of facial coverage/masks, at least in BC, as one participant noted: *“if you look back, the use of masks in indoor spaces like stores was suggested by the Health Minister, but not mandatory. Now, look at what happened: you will not be allowed inside a store if you do not have face coverage”* (29-year-old female front desk staff with 7 years of experience).

When asked about the visual representation and need to rearrange Fig. 1, many participants suggested compressing or decreasing the amount of words pertaining to the enablers and barriers. As we were told, *“those aspects of the provider characteristics are key, yes, but too many words there … they sort of overshadow the model”* (33-year-old female dentist with 5 years of experience, currently working part-time). More specifically, one participant suggested *“moving that information [provider characteristics and social and environmental context] to the top and bottom, almost like overseeing the actual model if you will”* (28-year-old dental hygienist with 4 years of experience). Many patients did not agree with the colour-coded scheme to show positive or negative ideas, as conveyed by a 42-year-old male patient currently seeing an oral health care provider: *“I mean, take media for example, it can be a good thing [green] when it is based on science, but a bad thing [red] when based on fake news.”*

**Fig. 3:**
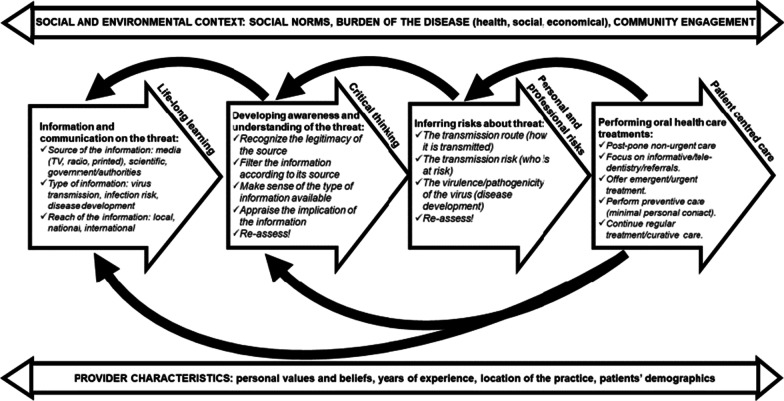
A modified preparedness model for the provision of oral health care during an unfolding health threat

With the suggestions provided during the interactions, a modified portrayal of the model was developed (Fig. 3) and sent back to all participants via email. Participants were asked to give feedback on the new portrayal, from its spatial representation to the content, as a member-checking exercise. In total, 27 participants responded; while most simply agreed with the portrayal there was some constructive feedback, including:

“The new model is much clearer, and the back-and-forth flow of information is shown by the multiple arrows, together with the top and bottom double-sided arrows showing continuity” (25-year-old male patient who has not resumed oral health care since the beginning of the pandemic).

“it is a new portrayal, yes … the text inside the now larger arrows illustrates the importance of those things, particularly the life-long learning that we all have to embrace and not just wait for things to happen” (51-year-old female dentist with 25 years of experience).

But for at least one participant, the modified portrayal was not as professional-looking as the original one: *“well, it does not look as professional as the first one…I mean, I would try making this model look like the first one, graphically speaking”* (37-year-old female dental hygienist currently working 3 days a week). Lastly, 21 participants suggested the model could represent any disease or infection, not just COVID-19. We heard from a currently working 35-year-old female dental assistant that *“I know we are in this pandemic now, but shouldn’t this model be used in any other similar event? I mean, knock on wood, but I think this will not be the last pandemic we have”.*

## Discussion

The aim of this study was to appraise a recently developed preparedness model to aid in decision-making for the provision of oral health care during a threat such as the COVID-19 pandemic from the perspectives of oral health care providers, administrators/staff, and patients. Such preparedness requires the development of communication plans and collaboration, while remaining flexible and adaptive to changes in disease development. In agreement with our previous findings [5, 6], the lack of a preparedness model to assist in understanding the threat posed by the COVID-19 pandemic likely led to uncertainties and social unease.

According to the participants, the proposed model should be used not only for COVID-19, but for any other outbreak. The use of a preparedness model is not new, however. From natural disasters [40] to terrorist attacks [41] and disease outbreaks [17, 42], preparedness activities have been suggested as a response to threats while trying to understand their impact on individuals and society at large. This is the goal of Fig. 3, given that the existing literature on preparedness and oral care is limited and mostly focus on surveys about the impact of COVID-19 [16] readiness to attend to medical emergencies [20, 21] and the willingness to see certain patients [22]. This study fills a gap in our current understanding of preparedness and oral care that might be applicable to future serious events.

The theme of life-long learning is not new in health literature. According to Panda and Desbiens, *“lifelong learning … provide*[s] *timely, efficient, and state-of-the-art patient care … where knowledge …* [is] *rapidly changing”* (page 562) [43], which is indeed the case for knowledge surrounding COVID-19. It should also be an integral part of maintaining health professionals' competence, as the onus to learn rests on their shoulders (as was mentioned). Continued learning [44, 45] can take many forms, from retraining courses to reading professional journals for credible information [46]. Life-long learners must strive to provide evidence-based care that meets the needs of those they serve [47] particularly during a pandemic that still impacts some more than others [6]

One’s life-long learning journey [48] goes hand-in-hand with critical thinking to appraise information fairly. In an era of copious amounts of information, careful consideration must be taken to filter fact from fiction, especially related to COVID-19 [49]. As information is always evolving, the need to re-assess and re-evaluate what is known is highlighted in the model. Mask and face coverage, for example, was exemplified as evolving and ever-changing knowledge. In this context, critical thinking should prevail, particularly when the potential of the proposed intervention is not well understood [50]. Similarly, the saying that “the risks outweigh the benefits” has been questioned during the pandemic, including in regard to the implementation of lockdowns [51]. The proposed model in Fig. 2 focuses on the risks of SARS-CoV-2 transmission where it is unlikely that a patient would accept seeing a health care professional without a face mask and other PPE. In fact, despite oral health care providers being considered to be at the highest risk for the transmission of the virus [7–9], the introduction of infection control protocols and PPE following the HIV/AIDS crisis [5] may explain the allegedly lower level of SARS-CoV-2 infection among these professionals so far even if the virus is indeed airborne [10]. Still, risks must be properly inferred so that incorrect assumptions about transmission routes and who is at risk for transmission are avoided [5, 10].

Finally, patient-centred care emerged when participants discussed the different levels of oral health treatments available. Patient-centred care is not only about taking care of individuals with dignity and respect, but also involving them in all decisions about their health [52]. Hence, whenever there is a decision to postpone treatment or to continue regular care during a pandemic, the patient must be collaboratively brought into the discussion despite reluctance from some providers to share decision-making, as discussed by Apelian and colleagues [53]. Patient-centred care should also be the focus of tele-dentistry, despite posing challenges to building rapport, collaborative communication, and billing [54] as also discussed by some of our participants.

This study aimed at achieving rigour through reflexivity, data saturation, and member checking. Rigor attempted to ensure that the research design, method, and conclusions presented herein were clear, of public access, replicable, open to critique, and free of bias [55] as we have done. Reflexivity was discussed when we described the intersecting contextual relationships between the participants and the pandemic [56]. Saturation referred to the point during data collection where no new information was provided on the issue under investigation, and the data did indeed become repetitive within the 74 interviews; the number of interviews necessary to achieve saturation varies greatly depending on the scope of the study [57, 58]. More specifically, saturation was attained when no new themes emerged from coding the subsequent interviews about the model in light of the study objective. Member checking took place at different stages of the study, where participants were given the opportunity to read their transcripts, and/or the thematic analysis, and/or the final model [59].

Despite the findings, our study has several limitations. Although the number of participants was significant and we reached saturation, they do not represent all oral health care workers or patients in BC or Canada; therefore, generalisation of the findings needs to be done with caution. The four major themes presented in Fig. 3 are not meant to be exhaustive or to represent all the ideas shared during this study, further analysis is highly recommended. Future studies should also include a sample of oral health care providers, administrators/staff, and patients from other jurisdictions across Canada to elicit any context relevant changes or confirm the content and representation of the model. Another limitation is the fact that we were not able to confirm if the preparedness activities mentioned were actually performed. A full evaluation of the proposed preparedness model is warranted to assess its validity and reliability [60] for guiding the decision-making process to pause or continue provision of oral health care during a threat such as a pandemic or disease outbreak, even with the advent of vaccines. Other contexts in which this model can be used must be explored, including both public and private sectors, while enabling collaboration in the decision making process.

## Conclusions

This study appraised a recently developed preparedness model for the provision of dental care during an unfolding threat such as COVID-19 from the perspectives of dental professionals, administrators/staff, and patients. Thematic analysis led to four main themes: life-long learning, critical thinking, personal and professional risk, and patient-centred care. These themes were understood within provider characteristics and social and environmental contexts. Participants highlighted the need to continued focus on information and communication, developing awareness and understanding, inferring risks, and performing oral health care during a threat such as a pandemic or disease outbreak. The appraisal led to modifications into a new portrayal of the preparedness model. Future studies are warranted to evaluate the modified model for use in the event of another unfolding threat collaboratively with providers, patients, and stakeholders.

## Data Availability

Due to the sensitivity of the information shared during the interviews, the data (interview transcripts) cannot be made available. The findings from the literature review used to develop our arguments are included in this published article. The full list of codes and categories can be retrieved by contacting the corresponding author.

## Abbreviations

AIDS: Acquired Immune Deficiency Syndrome
BC: British Columbia
COVID-19: Coronavirus Disease 2019
HIV: Human immunodeficiency virus
PPE: Personal protective equipment
SARS-CoV-2: Severe acute respiratory syndrome–coronavirus-2
UPs: Universal precautions
WHO: World Health Organization
